# Chest computed tomography performed on admission helps predict the severity of smoke-inhalation injury

**DOI:** 10.1186/cc12740

**Published:** 2013-05-25

**Authors:** Hitoshi Yamamura, Shinichiro Kaga, Kazuhisa Kaneda, Yasumitsu Mizobata

**Affiliations:** 1 Department of Critical Care Medicine, Graduate School of Medicine, Osaka City University, 1-4-3 Asahimachi, Abenoku, Osaka City, Osaka 545-8585, Japan

**Keywords:** smoke-inhalation injury, pneumonia, bronchial-wall thickness

## Abstract

**Introduction:**

Smoke-inhalation injury is a major cause of mortality in burn patients, and therefore, it is important to determine accurately the severity of such injuries in these patients. The objective of this study was to evaluate whether chest computed tomography (CT) can be used for detecting early predictors of severity and complications of smoke-inhalation injury.

**Methods:**

We evaluated 37 patients who had sustained smoke-inhalation injuries and had undergone chest CT within a few hours of admission to a hospital. Bronchoscopy was performed according to a standardized protocol within 12 hours of admission in all smoke-inhalation injury patients. Bronchial-wall thickness (BWT) was measured 2 cm distal from the tracheal bifurcation with CT images, and the following data were collected: total number of ventilator days, duration of intensive care unit (ICU) stay, pneumonia development, and patient outcome.

**Results:**

The mean age of the patients was 63 ± 18 years (range, 22 to 87 years), 31 (83.8%) of the patients were men, and the mortality rate was 10.8%. The causes of death in these patients were smoke inhalation (*n *= 1), hemorrhage (*n *= 1), and other factors resulting in sepsis (*n *= 2). The initial carboxyhemoglobin level was 13% ± 14% (range, 1% to 50%). No significant correlation was found between bronchoscopic scoring and clinical factors. However, significant correlations were noted between admission BWT and development of pneumonia (R^2 ^= 0.41; *P *< 0.0001) and total number of ventilator days (R^2 ^= 0.56; *P *< 0.0001) and ICU-stay days (R^2 ^= 0.17; *P *= 0.01). Receiver operating characteristic curve analysis showed that an admission BWT cutoff value of >3.0 mm predicted pneumonia development with a sensitivity of 79%, specificity of 96%, positive predictive value of 91%, and negative predictive value of 88%.

**Conclusion:**

BWT measured by using the chest CT scans obtained within a few hours of admission was predictive of the total number of ventilator days and ICU-stay days and the development of pneumonia in patients with smoke-inhalation injuries.

## Introduction

Smoke-inhalation injury (SII) is a major cause of mortality in burn patients because it can trigger pneumonia and acute respiratory distress syndrome (ARDS) [1]. It is important that SII be diagnosed early, thus facilitating an appropriate treatment strategy. Accurately diagnosing the severity of SII is difficult, and several methods, including bronchoalveolar lavage [2], computed tomography (CT) of the chest to measure the area of patchy ground-glass appearance [3], and bronchoscopic findings [4,5] have been reported. Conventional methods are not only technically difficult to perform but also have limitations such as low sensitivity and specificity. Accurate diagnosis of the severity of SII is important for predicting the risk of pneumonia development and the duration of mechanical ventilation that may be required. The objective of this study was to evaluate whether chest computed tomography (CT) can be used for detecting early predictors of severity and complications of SII.

## Materials and methods

### Patient selection

Thirty-seven patients admitted to a Critical Care Unit in Osaka City University Hospital were enrolled in this prospective study. Patient selection was performed sequentially. These patients had sustained SII and had undergone chest CT within a few hours of admission. Approval of our institutional Ethics Committees for the study protocol was obtained, and written informed consent was obtained from each patient or the patient's family before inclusion in the study. SII was suspected on the basis of smoke exposure within a confined space or soot at the nares, pharynx, or larynx. Exclusion criteria were as follows: age younger than 18 years, chronic obstructive pulmonary disease, malignancy, or burn surface area >20%. We evaluated, as the control, 10 volunteers who were all undergoing evaluation by medical examination. Findings from chest CT scans of the volunteer-group patients were interpreted by a radiologist as being within normal range.

### Measurements

The clinical data recorded included age, sex, total surface area of the burn, associated injury, inhalation injury grade by bronchoscopy, initial arterial blood gas analysis, initial plasma carboxyhemoglobin (CO-Hb), time between smoke inhalation and CT, initial 24-hour fluid volume, total number of ventilator days, duration of intensive care unit (ICU) stay, pneumonia development, and patient outcome. We defined pneumonia as consolidation on the chest radiograph film, body temperature of >38°C or <36°C, white blood cell count of ≥12,000 cells/mm^3 ^or ≤4,000 cells/mm^3^, and positive culture of sputum, except for normal respiratory/oral flora or mixed respiratory/oral flora or equivalent. Extubation criteria were adequate mentation and the capacity to maintain adequate arterial partial pressure of oxygen/inspired oxygen fractions arterial partial pressure of oxygen to inspired oxygen fraction [PaO_2_/F_I_O_2_] ratio >200) provided by using simple oxygen devices (F_I_O_2 _<0.4 and with low levels of positive end-expiratory pressure (PEEP) of <5 cm H_2_O).

### Bronchoscopy

Bronchoscopy was performed according to a standardized protocol within 12 hours of admission in all SII patients. The degree of bronchial mucosal status was evaluated by using a standardized bronchoscopic scoring system based on the Abbreviated Injury Score (AIS) criteria, as previously published [6]. This scoring was graded into five categories (0, no injury; 1, mild; 2, moderate; 3, severe; and 4, massive injury) (Table 1). Several emergency room physicians performed the bronchoscopies, and these images were recorded and evaluated later by two specific respiratory physicians.

**Table 1:** Abbreviated Injury Score (AIS) for bronchoscopic gradation of inhalation injury

Grade	Findings
Grade 0 (no injury)	Absence of carbonaceous deposits, erythema, edema, bronchorrhea, or obstruction
Grade 1 (mild injury)	Minor or patchy areas of erythema, carbonaceous deposits in proximal or distal bronchi (any or combination)
Grade 2 (moderate injury)	Moderate degree of erythema, carbonaceous deposits, bronchorrhea, with or without compromise of the bronchi (any or combination)
Grade 3 (severe injury)	Severe inflammation with friability, copious carbonaceous deposits, bronchorrhea, bronchial obstruction (any or combination)
Grade 4 (massive injury)	Evidence of mucosal sloughing, necrosis, endoluminal obliteration (any or combination)

### Computed tomography

**Figure 1:**
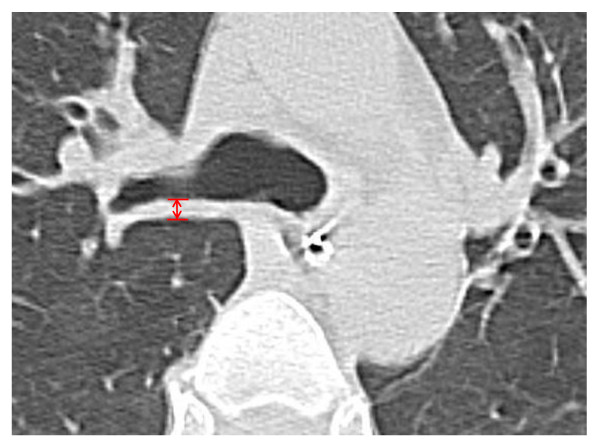
**Indices of bronchial wall thickness on HRCT scan**. Measurement position of bronchial-wall thickness (arrows). HRCT, helical high-resolution computed tomography.

CT images were obtained on a SOMATOM CT system (Siemens AG, Forchheim, Germany). All CT images were noncontrast-enhanced 1.5-mm helical scans. Scans were obtained at the end of inspiration by using a 1-second scan time and a high spatial frequency. Bronchial-wall thickness (BWT) was measured 2 cm distal from the tracheal bifurcation (Figure 1). The BWT was measured at three time points (that is, at admission, 24 hours after admission, and 7 days after admission). Measurement of BWT was performed after the patients were discharged. The BWT was measured by a radiologist who was blinded to the clinical findings and patient data.

### Statistical analysis

Data are shown as mean ± SD. Patient demographic data and outcomes were assessed for normality, and parametric or nonparametric tests were performed, as appropriate. Linear regression or χ^2 ^analysis was used to calculate statistical significance. A difference between observed variables was considered statistically significant when *P *< 0.05. Statistical analyses were performed with SPSS Software, Version 16.0 (SPSS Inc., Chicago, IL, USA).

## Results

A total of 33 patients survived, and the mortality rate was 10.8%. The causes of death were smoke inhalation (*n *= 1), hemorrhage (*n *= 1), and other factors resulting in sepsis (*n *= 2) (Table 2). The trauma patient had a retroperitoneal hemorrhage due to pelvic fracture, cutaneous burn, and head injury (subarachnoid hemorrhage and brain contusion). Transcatheter embolization was performed to treat retroperitoneal hemorrhage; however, the patient's disseminated intravascular coagulation progressed, and the hemorrhage was uncontrollable. Death in the two patients with sepsis was the result of pneumonia in one patient and septic shock from wound sepsis in the other patient. Twelve patients did not require intubation and mechanical ventilation. Five of eight body-surface burn patients needed operative treatment. The mean hospital stay of all patients was 17 ± 15 days, and that of the dead patients was 10 ± 6 days.

**Table 2:** Patient demographics and characteristics

Characteristic	Subjects (*n *= 37)
Age, mean (SD), years	63 (18)
Sex, *n *(%)	
Female	6 (16.2)
Male	31 (83.8)
Initial BP (mm Hg)	
Initial CO-Hb, mean (SD), %	13 (14)
Initial BE, mean (SD)	-3.5 (6.1)
Interval between SII and CT examination, mean (SD), minutes	98 (36)
Interval between admission and CT examination, mean (SD), minutes	79 (37)
Initial 24-hour fluid volume, mean (SD), milliliters	5,684 (4,092)
	
Associated injury	
Burns	8
TBSA, mean (SD), %	11.5 (8.7)
3^rd ^degree burn, mean (SD), %	10.5 (9.0)
Trauma	1

BE, base excess; BP, blood pressure; CO-Hb, carboxyhemoglobin; CT, computed tomography; SD, standard deviation; SII, smoke-inhalation injury; TBSA, total burn surface area.

No significant correlation was found between bronchoscopic scoring and clinical factors. However, significant correlations occurred between admission BWT and development of pneumonia and total number of ventilator days and ICU stay days (Table 3). The BWT of early-discharge patients, whose hospital stays were <5 days, was 1.75 ± 1.16. Receiver operating characteristic curve analysis showed that admission BWT cutoff value of >3.0 mm predicted pneumonia development with a sensitivity of 79%, specificity of 96%, positive predictive value of 91%, and a negative predictive value of 88%. Receiver operating characteristic curve analysis also showed that a bronchoscopic score of more than 2.0 had a sensitivity of 50%, specificity of 70%, PPV of 50%, and NPV of 70% for predicting pneumonia development.

**Table 3:** Correlations between BWT or bronchoscopic scoring and clinical indices

	Admission BWT			Bronchoscopic scoring		
	
	R^2^	Regression coefficient	*P*	R^2^	Regression coefficient	*P*
Duration SII and CT	0.003	1.45	0.75	0.007	92.28	0.63
P/F ratio	0.031	-21.70	0.35	0.033	454.80	0.03
Infusion volume, 24 hours	0.081	952	0.09	0.005	6,040	0.67
Balance, first 24 hours	0.055	822	0.18	0.02	4,552	0.42
% TBSA	0.315	-3.91	0.19	0.255	5.95	0.25
Development of pneumonia	0.407	0.25	<0.0001	0.05	0.25	0.18
Ventilation days	0.561	3.26	<0.0001	0.08	1.68	0.16
ICU-stay days	0.172	2.64	0.01	0.01	5.52	0.48
Hospital days	0.097	4.20	0.06	0.013	14.10	0.51
Outcome	0.071	-0.07	0.11	0.006	0.86	0.65

BWT, bronchial-wall thickness; CT, computed tomography; ICU, intensive care unit; % TBSA, % total burn surface area; SII, smoke-inhalation injury.

Change in BWT at the three time points investigated (that is, at admission, 24 hours, and 7 days after admission) is shown in Table 4. The BWT could be measured in 29 of the 37 patients at all three time points. In eight patients, CT was not performed at 24 hours or 7 days after admission because of early discharge (within 4 days; *n *= 6) or respiratory or circulatory compromise within 24 hours of admission (*n *= 2). In the early-discharge patients, informed consent for examination by follow-up CT at 24 hours after admission could not be obtained. All patients exhibited increased BWT on admission, which decreased by 24 hours after admission. Twenty-six patients had a BWT of <2.0 at 7 days after admission. Three patients had a BWT of >2.0 at 7 days after admission, and in all of these patients, pneumonia developed, with a mean hospital stay of 17 ± 1 days. The BWT of the normal volunteers was 1.0 ± 0.6.

**Table 4:** Change in BWT

	Admission	24 hours	7 days
Total BWT			
m (SD)	2.45 (1.26)	3.40 (1.20)^a^	1.49 (0.64)^ab^
N	37	29	29
BWT of SII alone			
m (SD)	2.43 (1.26)	3.18 (1.00)^a^	1.52 (0.68)^ab^
Number	29	23	23
BWT of SII with cutaneous burn			
m (SD)	2.53 (1.34)	4.16 (1.71)^a^	1.42 (0.68)^ab^
Number	8	6	6

BWT, bronchial-wall thickness; SD, standard deviation; SII, smoke-inhalation injury.

^a^*P *< 0.001 versus admission BWT; ^b^*P *< 0.001 versus 24 hours BWT. BWT values in the 7-days-after group were significantly lower than those in the admission (*P *< 0.001) and 24-hours-after (*P *< 0.001) groups. BWT values in the 7-days-after group with SII alone and SII with cutaneous burn were significantly lower than those in the admission (*P *< 0.001) and 24-hours-after (*P *< 0.001) groups.

**Figure 2:**
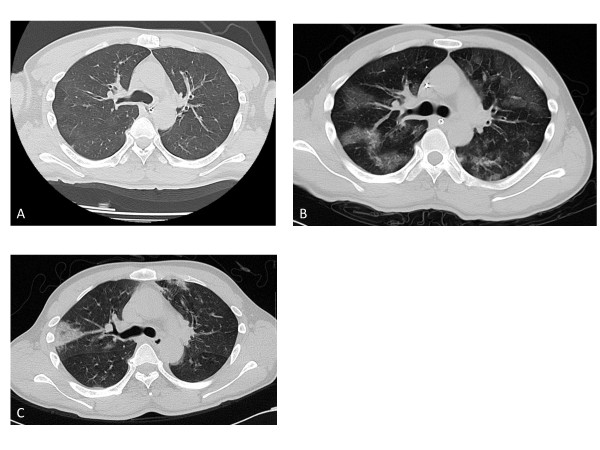
**A typical case of SII chest CT**. The BWT was 4.1 mm on admission **(A)**, 4.4 mm after 24 hours **(B)**, and 2.8 mm after 7 days **(C)**. He had associated pneumonia and remained in the ICU for 14 days. SII, smoke-inhalation injury; CT, computed tomography; BWT, bronchial-wall thickness; ICU, intensive care unit.

A typical case of smoke-inhalation injury with increased BWT is shown in Figure 2. The patient had SII after being rescued from a house fire and was brought to our emergency department with an initial carboxyhemoglobin level of 50.3%. A chest CT obtained 0.5 hours after admission showed absolutely normal findings, with the exception of bilaterally thickened bronchial walls; BWT on admission CT was 4.1 mm (Figure 2A). The CT scan obtained 24 hours later showed a bilateral patchy ground-glass shadow, and the BWT was 4.4 mm (Figure 2B). The CT scan obtained on day 7 of hospitalization revealed only atelectasis and showed that the BWT had decreased to 2.8 mm (Figure 2C).

## Discussion

The need to predict accurately the severity of SII is important. Accurate determination of the severity of SII would substantially contribute to effective treatment. Parameters such as estimates of the volume of infusion, as well as assessment of the need for mechanical-ventilation management, the possibility of pneumonia development, and outcome may be necessary. The severity of SII in the early phase immediately after the incident has rarely been investigated [2-5,7,8]. To our knowledge, this study is the first to use chest CT to describe and evaluate the severity of SII in patients in the early phase after injury. Helical CT is a valuable tool for assessing airway wall thickness, and airway-lumen dimension, as it enables noninvasive, reproducible evaluation of the airways at the level of the bronchioles.

We measured the BWT at admission and found that it was of early predictive value with regard to the development of pneumonia, the total number of ventilator days, and ICU-stay days. Airway wall thickness was also thought to be due to toxic inhalation, suggesting inhalation injury to the middle to lower part of the airway. These findings were observed in the acute and subacute phases of the injury. In addition to reversible airway-wall thickening, many factors such as airway-wall edema, inflammation, mucus secretion, and bronchial smooth muscle spasms may contribute to airflow obstruction in SII.

SII was traditionally diagnosed by using a bronchoscope; however, this method could not quantify the severity of the SII. A bronchoscopic finding of inhalation injury is inherently subject to interrater differences, such as their perceptions of carbonaceous deposits, erythema, edema, bronchorrhea, or obstruction. A previous study reported that bronchoscopy findings are correlated with SII severity, and thus bronchoscopy can be used to detect upper- or large-airway problems; however, this technique does not enhance or predict the necessity of mechanical ventilation. Further, duration of intubation, level of PEEP, and survival appear to be independent of the initial bronchoscopic findings [4]. Another report suggested that AIS grading of inhalation injury correlates moderately with early perturbations in oxygenation, development of ARDS, prolonged ventilator dependence, and increased acute fluid-resuscitation needs [5].

The AIS criteria constitute a standardized bronchoscopic scoring system for the degree of inhalation injury. In our study, AIS score derived from bronchoscopic findings did not correlate with any clinical value such as development of pneumonia or total days of mechanical ventilation. Bronchoscopic findings were varied and included observations such as carbonaceous deposits, which may render it difficult to determine SII severity. Our results did not correlate with the clinical indices. However, bronchoscopy is useful in identifying and grading airway injury, performing bronchial toilet, retrieving specimens for culture, and detecting infection before clinical pneumonia is diagnosed.

Another novel finding of our study was the change in BWT over the clinical course of SII. In most cases, BWT increased after admission. The three patients who had a BWT of >2.0 mm at 7 days after admission developed pneumonia, and the mean number of days on mechanical ventilation in these patients was 9 (range, 4 to 18 days); their condition was more severe than that of the others. We measured BWT of normal volunteers as controls, and these values were all <2.0 mm.

Previously reported causes of bronchial wall thickening include bronchial asthma [9-13] and chronic obstructive pulmonary disease [14-17]. Many investigators have reported a thicker reticular basement membrane in patients with asthma than in healthy subjects. Previous studies have also shown that thickening of either the bronchial reticular basement membrane or the airway wall correlates with disease severity, a decrease in respiratory function, and airway hyperresponsiveness in patients with asthma [9-13].

Albright [2] reported that the method of assessing SII severity involves analyzing bronchoalveolar lavage fluid for leukocyte differentiation and the concentrations of specific cytokines, chemokines, and growth factors. This study assessing bronchoalveolar lavage fluid showed that a greater severity of inhalation injury is associated with a greater degree of alveolar neutrophilia, prolonged ventilator requirements, longer stay in both the intensive care unit and the hospital, and enhanced pulmonary inflammatory mediator production [2].

Other studies of SII using CT examination include the study of Clark *et al. *[18], who reported the superior sensitivity of CT scans in comparison to chest radiographs and xenon-133 lung scans for the detection of pulmonary lesions within 2 hours of severe SII in dogs. This study also reported that the potential utility of CT in the treatment of patients with SII is not limited to determination of the severity of the injury.

Several limitations to our study include the following. First, the patients in our study had a tendency toward lower or moderate SII severity. An inherent selection bias occurred: the candidates suspected of having very severe inhalation injury were chosen for enrollment. Second, our study had a relatively small sample size, which did not permit us to detect small differences in total ventilator days; thus, we should examine more cases in the future. Third, we were unable to link the development of pneumonia to another origin that could account for the possibility that aspiration of oral, pharyngeal, or gastroduodenal contents may lead to the development of pneumonia. However, our CT results were more predictive of the development of pneumonia as compared with bronchoscopic findings. Fourth, in regard to our exclusion criteria, the patients younger than 18 years were excluded because their BWT is thinner than that of patients older than 18 years. We conducted the study to evaluate patients with uniform BWT. Further, we excluded patients with a total burn surface area of >20% because we could not perform CT scanning safely because of the massive volume resuscitation required by such patients who are in respiratory failure and a hypovolemic state. However, our hospital did not admit any patients with >20% body-surface burns with SII during the study period.

## Conclusions

BWT measured by using chest CT scans obtained within a few hours of admission was predictive of the total number of ventilator days and ICU-stay days and the development of pneumonia in patients with SII. Our findings suggest that BWT of the airway appears in patients with SII and that these reversible changes of the airway wall may cause deterioration of respiratory function and refractory airway obstruction.

## Key messages

• The significant association between bronchial wall thickness on admission and development of pneumonia suggests that bronchial wall thickness may be a predictor of the severity of smoke-inhalation injury.

• A positive correlation between bronchial wall thickness on admission and total number of ventilator days is proven in smoke-inhalation-injury patients.

• Bronchial wall thickening in smoke-inhalation injury is a reversible change of the airway wall that may cause deterioration of respiratory function and refractory airway obstruction.

## Abbreviations

AIS: Abbreviated Injury Score; ARDS: acute respiratory distress syndrome; BE: base excess; BP: blood pressure; BWT: bronchial-wall thickness; CO-Hb: carboxyhemoglobin; CT: computed tomography; HRCT: helical high-resolution computed tomography; ICU: intensive care unit; PaO_2_/F_I_O_2: _arterial partial pressure of oxygen to inspired oxygen fraction ratio; PEEP: positive end-expiratory pressure; SII: smoke-inhalation injury; SD: standard deviation; TBSA: total burn surface area.

## Competing interests

The authors declare that they have no competing interests.

## Authors' contributions

HY made substantial contributions in data acquisition, patient care, and writing of the manuscript. MY contributed to the study design, statistical analysis, and interpretation of data. SK and YK equally made substantial contributions in data acquisition and patient care, as well as reviewing the manuscript. HY and MY critically revised the manuscript for important intellectual content. All authors read and approved the final manuscript.
